# WTAP‐Mediated m^6^A Modification of circSMOC1 Accelerates the Tumorigenesis of Non‐Small Cell Lung Cancer by Regulating miR‐612/CCL28 Axis

**DOI:** 10.1111/jcmm.70207

**Published:** 2024-12-04

**Authors:** Xun‐Xia Zhu, Xiao‐Yu Chen, Li‐Ting Zhao, Xue‐Lin Zhang, Yi‐Ou Li, Xiao‐Yong Shen

**Affiliations:** ^1^ Department of Thoracic Surgery Huadong Hospital, Fudan University Shanghai China; ^2^ Department of Nursing Huadong Hospital, Fudan University Shanghai China; ^3^ Department of Critical Care Medicine Tongren Hospital, Shanghai Jiaotong University School of Medicine Shanghai China

**Keywords:** circSMOC1, growth, miR‐612, NSCLC, WTAP

## Abstract

Accumulating evidence reveals that deregulated N6‐methyladenosine (m^6^A) RNA methylation and circular RNAs (circRNAs) are required for the tumorigenesis of non‐small cell lung cancer (NSCLC). We aimed to uncover the underlying mechanisms by which WTAP‐mediated m^6^A modification of circRNA contributes to NSCLC. The differentially‐expressed circRNAs were identified by a circRNA profiling microarray. The association of circSMOC1 with clinicopathological features and prognosis in patients with NSCLC was estimated by fluorescence in situ hybridization. WTAP‐mediated m^6^A modification of circRNA was validated by RNA immunoprecipitation (RIP) and methylated RIP (MeRIP) assays. The role of circSMOC1 in NSCLC cells was validated by in vitro functional experiments and in vivo tumorigenesis models. CircSMOC1‐specific binding with miR‐612 was verified by RIP, luciferase gene report and RT‐qPCR assays. The effect of circSMOC1 and/or miR‐612 on CCL28 expression was detected by RT‐qPCR and Western blotting analysis. We found that the expression levels of circSMOC1 were elevated in NSCLC tissues and associated with TNM stage and poor survival in patients with NSCLC. Knockdown of circSMOC1 impaired the tumorigenesis of NSCLC in vitro and in vivo, whereas restored expression of circSMOC1 displayed the opposite effect. Furthermore, WTAP was upregulated in NSCLC and mediated m^6^A modification of circSMOC1 and circSMOC1 abolished WTAP knockdown‐caused tumour‐suppressive effects. Then, circSMOC1 acted as a sponge of miR‐612 to upregulate CCL28 and miR‐612 inhibitors abrogated circSMOC1 knockdown‐caused anti‐proliferation effects and CCL28 downregulation in NSCLC cells. Knockdown of CCL28 inhibited cell proliferation and invasion and counteracted miR‐612 inhibitor‐caused tumour‐promoting effects. Our findings unveil that WTAP‐mediated m^6^A modification of circSMOC1 facilitates the tumorigenesis of NSCLC by regulating the miR‐612/CCL28 axis.

## 1 Introduction

Lung cancer is one of the most common malignancies and ranks the second cancer‐related mortality worldwide [1]. Despite tremendous progress made in cancer therapeutics, non‐small cell lung cancer (NSCLC) as a pivotal constituent of lung cancer still possesses poor prognosis in advanced cases owing to tumour invasiveness and metastasis [2]. Increasing evidence unveils that dysregulated noncoding RNAs is involved in the prognosis and development of NSCLC [3, 4]. Therefore, comprehensive understanding of the molecular mechanism underlying NSCLC progression is essential for effective therapeutic strategies.

Circular RNAs (circRNAs) have been considered as a subgroup of noncoding RNAs and have characteristics of RNA stability due to covalent closed loop structure and RNase R resistance [5]. A handful of studies unveil that circRNAs act in cancer, including NSCLC, by regulating RNA‐binding proteins [6], sponging miRNAs [7, 8] and affecting protein translation [9, 10]. It is reported that circFGFR1, circSATB2 and hsa_circ_0003222 function as oncogenes by sponging miR‐381‐3p and miR‐527 [8, 11, 12], whereas circNDUFB2, circPTPRA and hsa_circ_0008305 act as tumour suppressors by sponging miR‐96‐5p and controlling TIF1γ [13, 14, 15]. Until now, the functional role of hsa_circ_0032363 (circSMOC1) in NSCLC needs further investigations.

It is generally acknowledged that microRNAs (miRNAs) act by post‐transcriptional regulation of targets involved in cancer progression. miR‐612 is downregulated in hepatocellular carcinoma (HCC) [16, 17], colorectal cancer (CRC) [18], cervical cancer [19], predicts favourable survival in patients with HCC [16] and represses cell growth and metastasis [16, 18, 19]. In addition, miR‐612 can be sponged by circETFA and hsa_circ_0001649 to impair HCC tumorigenesis and metastasis [20, 21].

N6‐methyladenosine (m^6^A) is one of the most common chemical modifications in eukaryotic mRNAs and m^6^A RNA methylation acts a crucial role in NSCLC [22]. It has been revealed that deregulation of m^6^A methyltransferase METTL3 [23], demethylase ALKBH5 [24] and m^6^A recognition proteins YTHDF1/2 [25, 26] is involved in the progression of NSCLC. Wilms tumour 1‐associated protein (WTAP) as an m^6^A writer drives hepatocellular carcinoma (HCC) progression [27] and mediates m^6^A modification of lncRNA DIAPH1‐AS1 to enhance nasopharyngeal carcinoma metastasis [28].

It has been reported that circ0008399 can interact with WTAP to facilitate assembly of m^6^A methyltransferase complex and cisplatin resistance in bladder cancer [29]. However, little knowledge is known about how WTAP‐mediated m^6^A modification of circRNA contributes to NSCLC. In the present study, we discovered a novel hsa_circ_0032363 (circSMOC1) in NSCLC and found that upregulation of circSMOC1 was associated with TNM stage and poor survival in patients with NSCLC. Further investigations revealed that WTAP‐mediated m^6^A modification of circSMOC1 promoted the tumorigenesis of NSCLC by regulating the miR‐612/CCL28 axis, providing a promising prognostic factor for NSCLC.

## 2 Materials and Methods

### 2.1 Clinical Samples

515 cases of lung adenocarcinoma (LAC) tissue samples and 59 pair‐matched LAC samples were collected from The Cancer Genome Atlas database (http://xena.ucsc.edu/getting‐started/). A tissue microarray (XT17‐002) including 80 pairs of NSCLC samples and 10 pairs of LAC tissue samples stored at −80°C were provided by Shanghai Outdo Biotechnology (Shanghai, China). Our study protocol was approved by the Ethics Committee of Huadong Hospital Affiliated to Fudan University.

### 2.2 Bioinformatic Analyses

A circRNA microarray was employed to screen the differentially‐expressed circRNAs between NSCLC and adjacent tissues and the data could be downloaded from the Gene Expression Omnibus (GEO) database (https://www.gcbi.com.cn/gclib/html/index). The miRNAs which can be sponged by circRNA were identified by the miRbase dataset (https://www.mirbase.org/index.shtml). The targets of miR‐612 were identified by the TargetScanHuman7.2 database (https://www.targetscan.org/vert_72/).

### 2.3 Fluorescence In Situ Hybridization (FISH)

The probe sequence for hsa_circ_0032363 (circSMOC1, 5′‐CACCACTGACATGGTT CAGG‐3′) were used to analyse the cellular location and expression levels of circSMOC1 (green fluorescent signal) in NSCLC tissue samples. The detailed description of FISH analysis was executed as previously reported [11].

### 2.4 RNA Extraction and Real‐Time Quantitative PCR (RT‐qPCR)

Total RNA was acquired by an RNA extraction kit (QIAGEN) and cDNA was synthesised by a reverse transcription kit (Promega, Madison, USA) according to the manufacturer's instructions. PCR was conducted for circSMOC1 and CCL28 amplification using the SYBR Green Master Mix and for miR‐612 using specific TaqMan probes (Thermo Fisher Scientific). After the reactions were finished, the relative mRNA levels of circSMOC1, miR‐612 and CCL28 were calculated using the $2^{-\Delta \Delta C_{t}}$. The primer sequences used are shown in Table S1.

### 2.5 Western Blotting

Cells were lysed by RIPA buffer (P0013B, Beyotime). The supernatants were resolved in SDS‐PAGE and transferred onto polyvinylidene fluoride (PVDF) membranes (Millipore), incubated with anti‐WTAP (10200‐1‐AP, Proteintech), anti‐caspase‐9 (AF6348, Affinity), anti‐cleaved caspase‐9 (AF5240, Affinity), anti‐caspase‐7 (DF6441, Affinity), anti‐cleaved caspase‐7 (AF4023, Affinity), anti‐caspase‐3 (DF6020, Affinity), anti‐cleaved caspase‐3 (AF7022, Affinity), anti‐CCL28 (N3C3, GeneTex) and anti‐GAPDH (AB‐P‐R 001) overnight at 4°C. Protein bands were scanned by enhanced chemiluminescence (ECL).

### 2.6 Plasmid, shRNA and Cell Transfection

The circSMOC1 plasmids, lentivirus‐mediated shRNA targeting circSMOC1 (sh‐circSMOC1, 5′‐AGGTGGGAGATGACGGGTCTA‐3′), WTAP plasmids, si‐CCL28, miR‐612 mimics and inhibitors were purchased from GenePharma (Shanghai, China). The negative vector (NC) and sh‐NC were regarded as the control groups. PC‐9, 95D, A549 and H1299 cells were planted in 6‐well plates 24 h prior to sh‐circSMOC1 lentivirus or WTAP plasmid transfection with 50%–60% confluence according to the manufacture instructions.

### 2.7 Methylated RNA Immunoprecipitation (MeRIP) and RIP

The MeRIP m^6^A Kit (Merck Millipore) was used for the immunoprecipitation. Then, the m^6^A enrichment of mRNA was analysed by qPCR. RNA‐Binding Protein Immunoprecipitation Kit (Millipore) as well as anti‐Ago2 antibody (10686‐1‐AP, Proteintech) or anti‐m^6^A antibody (ABE572, Merck Millipore) were used for RIP assay according to the manufacture instructions.

### 2.8 Actinomycin D and RNase R Treatment

Transcription was blocked by the addition of 2 mg/mL Actinomycin D. Total RNA was incubated for 30 min at 37°C with 3 U/μg of RNase R (Epicentre Technologies, USA).

### 2.9 CCK‐8, Colony Formation, Transwell Assays, Flow Cytometry and IHC Analyses

These assays were performed as previously reported [11].

### 2.10 Dual‐Luciferase Reporter Assay

PC9 and 95D cells were seeded into 24‐well plates and PRL‐TK‐Luc report systems carrying WT or Mut 3′UTR of circSMOC1 and CCL28 were co‐transfected with miR‐612 mimic or inhibitor into PC9 and 95D cell lines. After the transfection for 48 h, luciferase activities were measured using a dual‐luciferase reporter system.

### 2.11 In Vivo Tumorigenesis Assay

The female Balb/C‐nu/nu nude mice (6–8 week old, 18–20 g) were purchased from the Shanghai Laboratory Animal Central. PC‐9 cells (2 × 10^6^) transfected with the stable transfection of sh‐SMOC1 or sh‐NC lentivirus were resuspended in 200 μL of sterile PBS and injected subcutaneously into the right flanks of mice. After 5 weeks, the mice were sacrificed and the tumour volume and weight were calculated. The animal experiments were approved by the Ethics Committee of Shanghai Huadong Hospital (2022JS‐042).

### 2.12 Statistical Analysis

Statistical analyses were processed with GraphPad Prism 7 (La Jolla, CA, USA). The values are expressed as the mean ± SD. Chi‐square, Student's *t* test and analysis of variance were used for comparisons between groups. Kaplan–Meier analysis was used to analyse the correlation of circSMOC1, miR‐612 and CCL28 in patients with NSCLC. Pearson Correlation Analysis was used to analyse the correlation of miR‐612 with circSMOC1 or CCL28. *p* < 0.05 was considered statistically significant.

## 3 Results

### 3.1 The Elevated Expression of circSMOC1 Was Associated With Poor Survival in Patients With NSCLC

**FIGURE 1:**
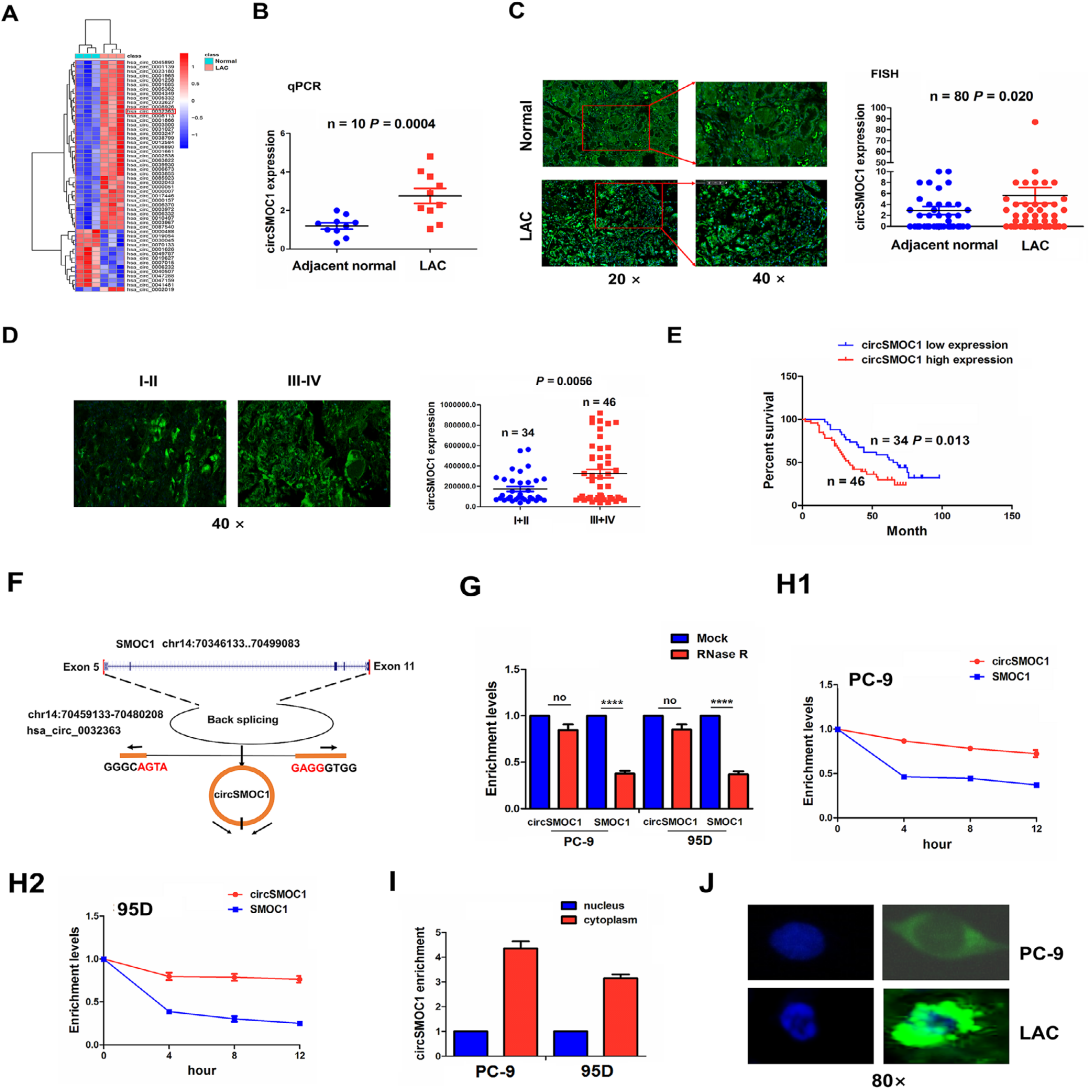
Upregulation of circSMOC1 was associated with poor survival in patients with NSCLC. (A) Hierarchical clustering of differentially expressed circRNAs between NSCLC and adjacent normal tissues. (B) RT‐qPCR analysis of the expression levels of circSMOC1 in 10 pairs of NSCLC tissues. (C) FISH analysis of the expression levels of circSMOC1 in 80 pair‐matched NSCLC tissues. (D) FISH analysis of the expression levels of circSMOC1 in NSCLC with stage I + II and stage III + IV. (E) Kaplan–Meier analysis of the association of circSMOC1 expression with overall survival in patients with NSCLC. (F) The genomic loci of circSMOC1. (G) RT‐qPCR analysis of the expression levels of circSMOC1 and SMOC1 after treatment with RNase R in PC‐9 and 95D cells. (H) RT‐qPCR analysis of the transcriptional stability of circSMOC1 and SMOC1 after treatment with Actinomycin D in PC‐9 and 95D cells. (I) RT‐qPCR and (J) FISH analysis of the location of circSMOC1 in NSCLC cells and tissues. Data shown are the mean ± SEM of three experiments. ****p < 0.0001.

The differentially‐expressed circRNAs between LAC and adjacent normal tissue samples were identified by the GEO database (GSE112214) and Heatmap indicated the top 36 upregulated and 18 downregulated circRNAs in LAC according to the FC > 2.0 and *p* < 0.01, among which hsa_circ_0032363 (circSMOC1) possessed a dramatical increase in LAC tissues (Figure 1A). The expression levels of circSMOC1 were examined by RT‐qPCR analysis, which indicated that circSMOC1 mRNA levels were markedly increased in 10 pairs of LAC tissue samples (Figure 1B; *p* = 0.0004). This result was further validated by FISH analysis in 80 pairs of LAC tissue samples (Figure 1C; *p* = 0.02). The increased expression of circSMOC1 was also shown in LAC with stage III–IV relative to stage I–II (Figure 1D; *p* = 0.0056). We then found that upregulation of circSMOC1 was associated with TNM stage (*p* = 0.008) in LAC patients (Table S2). Kaplan–Meier analysis unveiled that the patients with circSMOC1‐high expression harboured a shorter survival compared to those with circSMOC1‐low group (Figure 1E).

According to the annotation from Circular RNA Interactome, hsa_circ_0032363 (chr14:70459133–70480208) is derived from exon 5, 11 regions within the SPARC related modular calcium binding 1 (SMOC1) locus and termed as circSMOC1 (Figure 1F). After exposure to RNase R treatment, we measured the mRNA levels of circSMOC1 and linear SMOC1 in NSCLC cells and found that circSOMOC1 exerted a resistance to RNase R treatment as compared with linear SMOC1 in PC9 and 95D cells (Figure 1G). After PC9 and 95D cells were exposed to Actinomycin D treatment for 12 h, circSMOC1 produced an obvious stability as compared with its linear SMOC1 (Figure 1H). FISH analysis showed that circSMOC1 was predominantly localised in the cytoplasm of NSCLC cells (Figure 1I,J).

### 3.2 CircSMOC1 Drove NSCLC Cell Growth and Invasion but Blocked Cell Apoptosis

**FIGURE 2:**
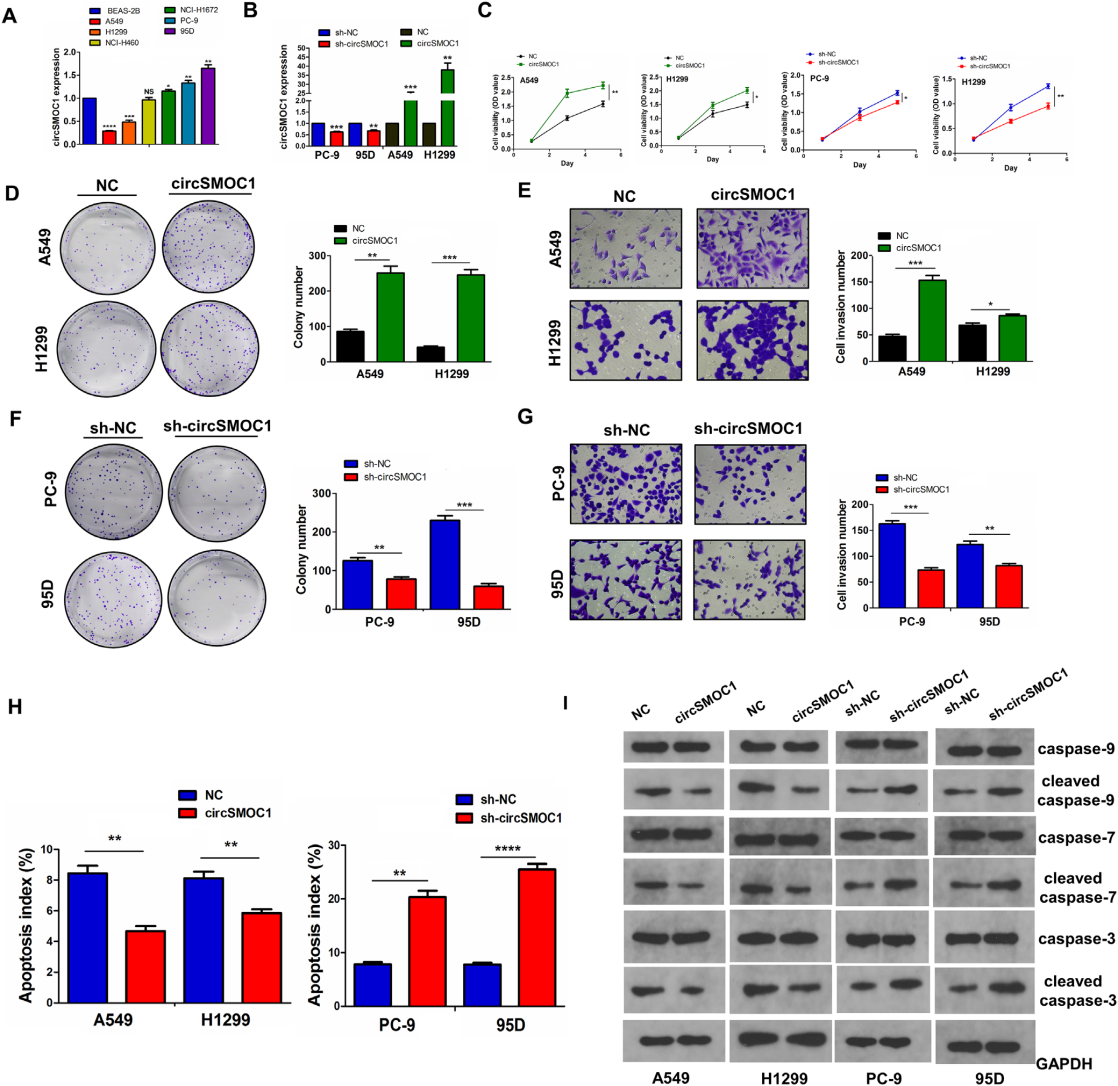
CircSMOC1 promoted NSCLC cell growth and invasion and blocked cell apoptosis. (A) RT‐qPCR analysis of the expression levels of circSMOC1 in various lung cancer cell lines. (B) RT‐qPCR analysis of the expression levels of circSMOC1 after the transfection with circSMOC1 plasmids in A549 and H1299 or sh‐circSMOC1 in PC‐9 and 95D cells. (C) CCK‐8 analysis of the cell viability after transfection with circSMOC1 plasmids in A549 and H1299 or sh‐circSMOC1 in PC‐9 and 95D cells. (D, F) Colony formation analysis of cell colony number after transfection with circSMOC1 plasmids in A549 and H1299 or sh‐circSMOC1 in PC‐9 and 95D cells. (E, G) Transwell analysis of cell invasion capabilities after transfection with circSMOC1 plasmids in A549 and H1299 or sh‐circSMOC1 in PC‐9 and 95D cells. (H) flow cytometry analysis of the effects of circSMOC1 overexpression or knockdown on cell apoptosis in NSCLC cells. (I) Western blotting analysis of the effects of circSMOC1 overexpression or knockdown on the protein levels of total or cleaved caspase ‐9, ‐7 and ‐3 in NSCLC cells. Data shown are the mean ± SEM of three experiments. *p < 0.05, **p < 0.01, ***p < 0.001, ****p < 0.0001.

We assessed the role of circSMOC1 in NSCLC cells and found that circSMOC1 harboured higher mRNA levels in PC‐9 and 95D cell lines but lower mRNA levels in A549 and H1299 cell liens (Figure 2A). Then, we established the circSMOC1‐overexpressed plasmids and the lentivirus‐mediated shRNA against circSMOC1. The overexpression of circSMOC1 could remarkably increase circSMOC1 mRNA expression in A549 and H1299 cell lines, whereas knockdown of circSMOC1 reduced its mRNA levels in PC‐9 and 95D cell liens (Figure 2B). Further experiments indicated that overexpression of circSMOC1 promoted cell viability, colony formation and cell invasion in A549 and H1299 cell lines (Figure 2C–E), but knockdown of circSMOC1 showed the opposite effects (Figure 2C,F,G). In addition, we investigated the effects of circSMOC1 on cell apoptosis by flow cytometry analysis, which indicated that overexpression of circSMOC1 reduced cell apoptosis in A549 and H1299 cells but knockdown of circSMOC1 induced cell apoptosis in PC‐9 and 95D cells (Figure 2H and Figure S1). Western blotting showed that overexpression of circSMOC1 decreased the protein levels of cleaved caspase‐9, ‐7 and ‐3 rather than their total protein levels in A549 and H1299 cells, while knockdown of circSMOC1 increased the protein levels of cleaved caspase‐9, ‐7 and ‐3 in PC‐9 and 95D cells (Figure 2I).

### 3.3 WTAP Mediated m^6^A Modification of circSMOC1 in NSCLC Cells

**FIGURE 3:**
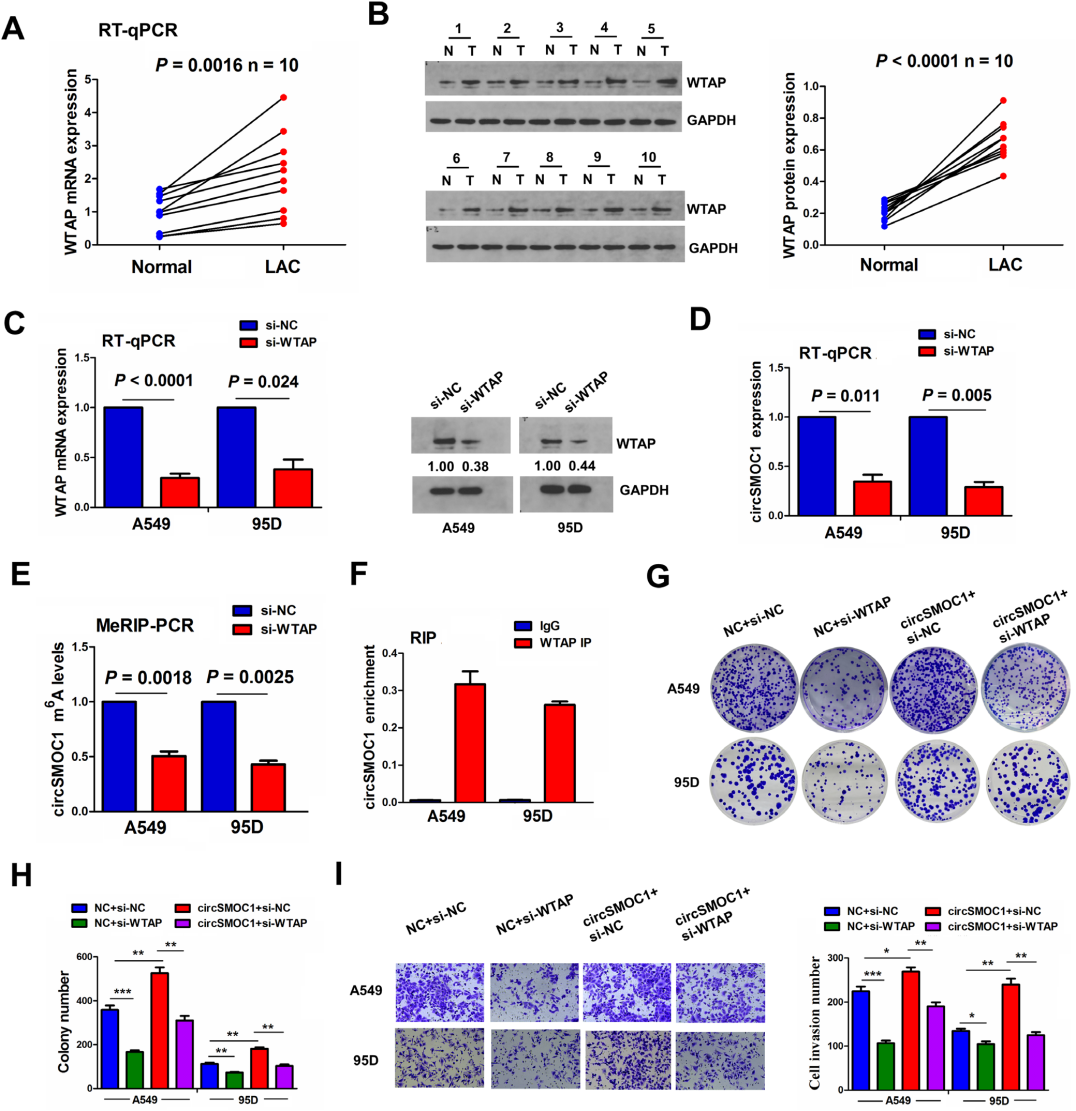
WTAP mediated m^6^A modification of circSMOC1 in NSCLC cells. (A) RT‐qPCR analysis of the expression levels of WTAP in 10 pairs of NSCLC tissues. (B) Western blotting analysis of the expression levels of WTAP in 10 pair‐matched NSCLC tissues. (C) RT‐qPCR and Western blotting analysis of the expression levels of WTAP after transfection with si‐WTAP into A549 and 95D cells. (D) RT‐qPCR analysis of the expression levels of circSMOC1 after transfection with si‐WTAP into A549 and 95D cells. (E) MeRIP analysis of the m^6^A levels of circSMOC1 after transfection with si‐WTAP into A549 and 95D cells. (F) RIP analysis of the interaction between circSMOC1 mRNA and WTAP protein in A549 and 95D cells. (G, H) Colony formation analysis of the cell colony abilities after co‐transfection with si‐WTAP and circSMOC1 into A549 and 95D cells. (I) Transwell analysis of the cell invasion abilities after co‐transfection with si‐WTAP and circSMOC1 into A549 and 95D cells. Data shown are the mean ± SEM of three experiments. *p < 0.05, **p < 0.01, ***p < 0.001.

To elucidate the mechanism of circSMOC1 high expression in NSCLC, we first analysed the m^6^A modification in circSMOC1 and found the interaction between circSMOC1 and WTAP by starBase3.0. Then, we measured the expression of WTAP in NSCLC by RT‐qPCR and Western blotting, which indicated that both of WTAP mRNA (Figure 3A) and protein levels (Figure 3B) were remarkably elevated in LAC tissues relative to the adjacent normal tissues. We constructed WTAP siRNA (si‐WTAP) and transfected it into NSCLC cells and found that knockdown of WTAP not only reduced the mRNA and protein levels of WTAP (Figure 3C) but also decreased the mRNA levels of circSMOC1 in A549 and 95D cells (Figure 3D). Furthermore, MeRIP showed that knockdown of WTAP dwindled the m^6^A levels of circSMOC1 in A549 and 95D cells (Figure 3E). A RIP assay was performed for WTAP protein in A549 and 95D cells, and the endogenous mRNA levels of circSMOC1 pulled down from WTAP‐expressed cells were predominantly enriched in the WTAP pellet relative to the IgG control group (Figure 3F). Functional assays revealed that knockdown of WTAP repressed the colony formation and cell invasion abilities and circSMOC1 abolished WTAP knockdown‐induced tumour‐suppressive effects in A549 and 95D cells (Figure 3G–I).

### 3.4 The miR‐612 Was Identified to Harbour Clinical Prognosis and Correlation With circSMOC1 Expression in NSCLC Tissues

**FIGURE 4:**
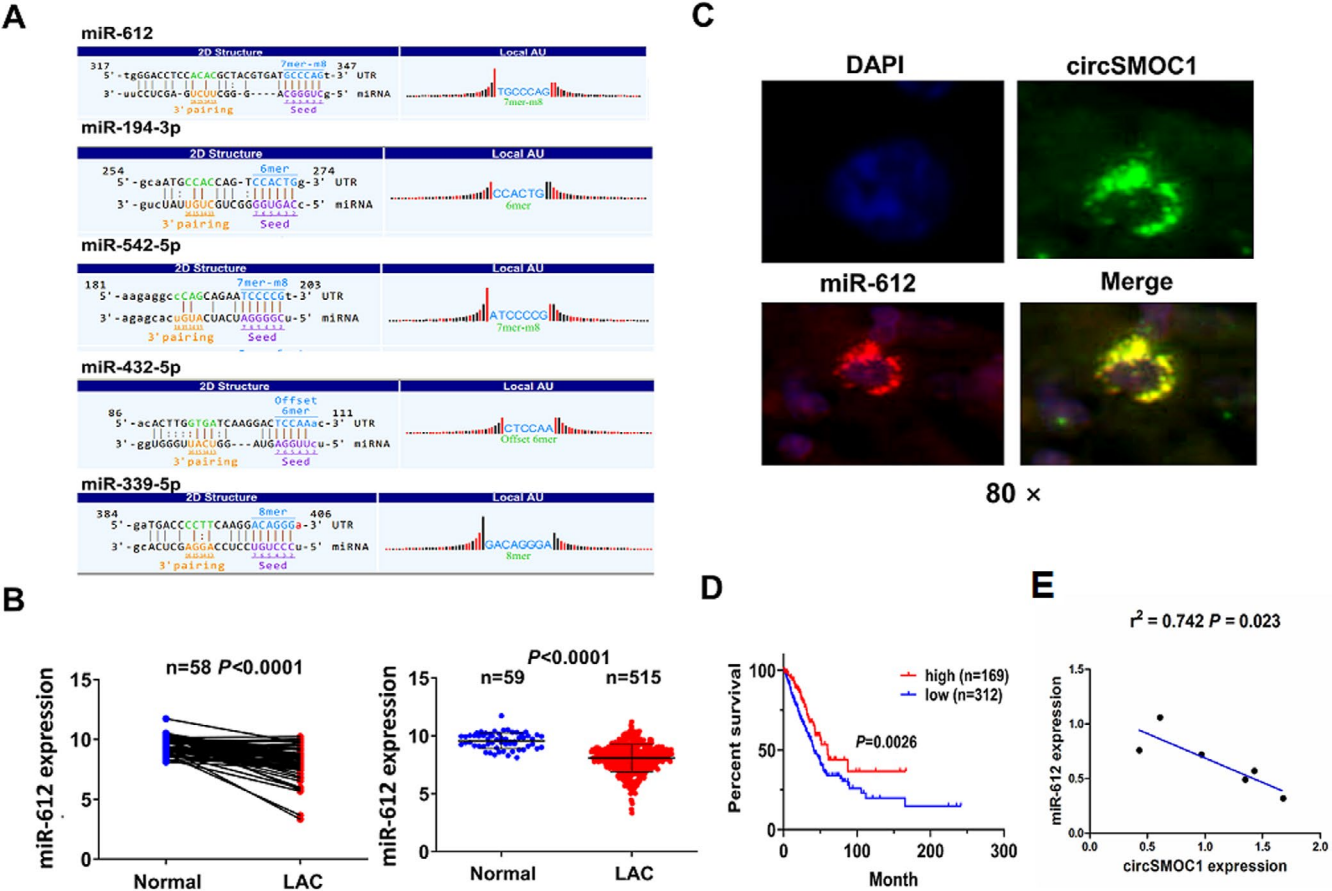
MiR‐612 harboured clinical prognosis and correlation with circSMOC1 expression in NSCLC. (A) Prediction of the binding sites between circSMOC1 and multiple miRNAs. (B) TCGA analysis of the expression levels of miR‐612 in pair‐matched and non‐matched NSCLC tissues. (C) FISH analysis of the cellular co‐location of circSMOC1 with miR‐612 in NSCLC tissues. (D) Kaplan–Meier analysis of the association of miR‐612 expression with overall survival in patients with NSCLC. (E) Pearson correlation analysis of the correlation of circSMOC1 with miR‐612 expression in lung cancer cell lines.

To corroborate the molecular mechanisms of circSMOC1 in NSCLC, we identified circSMOC1‐specific binding with miRNAs (miR‐612, miR‐194‐3p, miR‐542‐5p, miR‐432‐5p and miR‐339‐5p) (Figure 4A). Intriguingly, according to the TCGA data, only miR‐612 indicated a significantly decreased expression in pair‐matched and non‐matched NSCLC tissue samples (Figure 4B), whereas other miRNAs presented the increased expression levels (Figure S2). Cell localization analysis confirmed that circSMOC1 was co‐localised with miR‐612 in the cytoplasm of NSCLC tissue cells (Figure 4C). Then, we found that low expression of miR‐612 was related to advanced pathological stage (*p* = 0.002) and lymph node infiltration (*p* = 0.005) in patients with NSCLC (Table S3). Kaplan–Meier analysis uncovered that the patients with miR‐612‐low expression harboured a shorter survival when compared to those with miR‐612‐high group (Figure 4D). However, the patients with miR‐612‐low expression had no difference in tumour recurrence as compared with those with miR‐612‐high expression (Figure S3). Pearson correlation analysis displayed that miR‐612 had a negative correlation with circSMOC1 expression in NSCLC cells (Figure 4E).

### 3.5 CircSMOC1 Could Act as a Sponge of miR‐612 in NSCLC Cells

**FIGURE 5:**
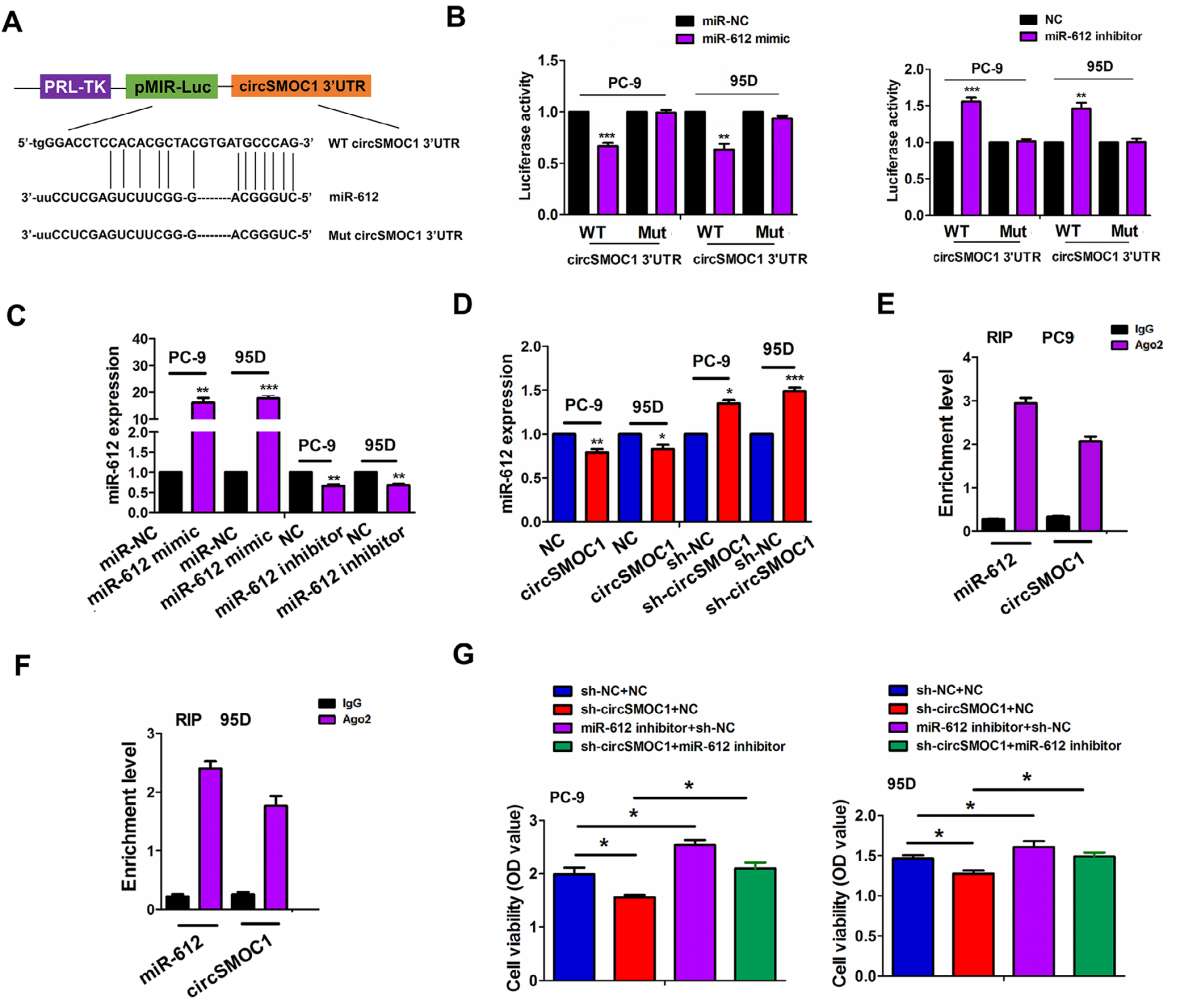
CircSMOC1 acted as a sponge of miR‐612 in NSCLC. (A) Schematic representation of the binding sites between miR‐612 and WT or Mut circSMOC1 3′UTR. (B) Analysis of luciferase activities of WT or Mut circSMOC1 3′UTR after co‐transfection with miR‐612 mimics/inhibitor and WT or Mut circSMOC1 3′UTR in PC‐9 and 95D cells. (C) RT‐qPCR analysis of the expression levels of miR‐612 after transfection with its mimics or inhibitor in PC‐9 and 95D cells. (D) RT‐qPCR analysis of the expression levels of miR‐612 after transfection with circSMOC1 plasmids or sh‐circSMOC1 in PC‐9 and 95D cells. (E, F) RIP analysis of the enrichment levels of circSMOC1 and miR‐612 pulled down from Ago2 protein in PC‐9 and 95D cells. (G) CCK‐8 analysis of the cell viability after co‐transfection with sh‐circSMOC1 and miR‐612 inhibitor in PC‐9 and 95D cells. Data shown are the mean ± SEM of three experiments. *p < 0.05, **p < 0.01, ***p < 0.001.

The binding sites of miR‐612 with WT or Mut circSMOC1 3′UTR could be shown in Figure 5A. To decipher whether miR‐612 can bind to circSMOC1 3′UTR, we co‐transfected WT or Mut circSMOC1 3′UTR and miR‐612 mimic or inhibitor into PC‐9 and 95D cell lines and found that miR‐612 mimics reduced the luciferase activities of WT circSMOC1 3′UTR, but miR‐612 inhibitors increased its luciferase activities (Figure 5B). However, miR‐612 exerted no impact on those of Mut circSMOC1 3′UTR. Further observations implied that miR‐612 mimic or inhibitor (Figure 5C) exhibited no effects on circSMOC1 mRNA expression (Figure S4), but circSMOC1 upregulation decreased miR‐612 mRNA expression while circSMOC1 downregulation promoted its mRNA expression in PC‐9 and 95D cell lines (Figure 5D). Furthermore, a RIP assay was employed for Ago2 protein in PC‐9 and 95D cells, and the endogenous expression of circSMOC1 and miR‐612 pulled down from Ago2‐expressed PC‐9 and 95D cells, measured by RT‐qPCR analysis was predominantly enriched in the Ago2 pellet relative to the IgG control group (Figure 5E,F). After co‐transfection with sh‐circSMOC1 and miR‐612 inhibitors into PC‐9 and 95D cells, we found that miR‐612 inhibitors facilitated the cell viability and abolished circSMOC1 knockdown‐caused anti‐proliferation effects (Figure 5G).

### 3.6 MiR‐612 Inhibitor Abrogated circSMOC1 Knockdown‐Caused Downregulation of CCL28

**FIGURE 6:**
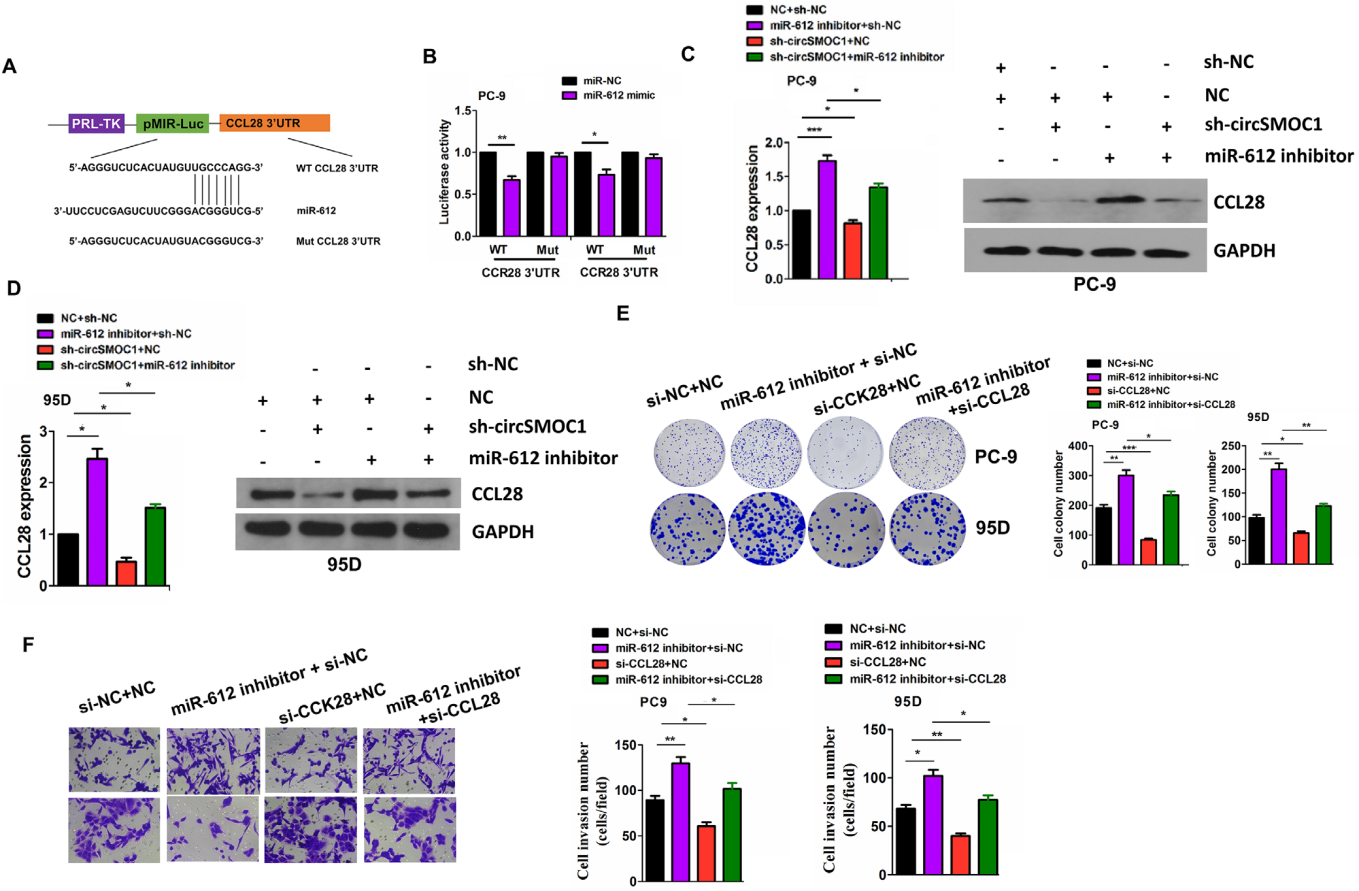
MiR‐612 inhibitor abolished circSMOC1 knockdown‐caused CCL28 downregulation in NSCLC. (A) Schematic representation of the binding sites between miR‐612 and WT or Mut CCL28 3′UTR. (B) Analysis of luciferase activities of WT or Mut CCL28 3′UTR after co‐transfection with miR‐612 mimics and WT or Mut CCL28 3′UTR in PC‐9 and 95D cells. (C, D) RT‐qPCR and Western blotting analysis of the expression levels of CCL28 after co‐transfection with sh‐circSMOC1 and miR‐612 inhibitor in PC‐9 and 95D cells. (E, F) Colony formation and transwell analysis of the cell colonies and invasion capabilities after co‐transfection with si‐CCL28 and miR‐612 inhibitor in PC‐9 and 95D cells. Data shown are the mean ± SEM of three experiments. Data shown are the mean ± SEM of three experiments. *p < 0.05, **p < 0.01, ***p < 0.001.

The targets of miR‐612 were further identified by Targetscan7.1. According to the cumulative weighted context score (−1.03), CCL28 was selected as the most beneficial target gene of miR‐612. The binding sites between miR‐612 and WT or Mut 3′UTR of CCL28 can be indicated in Figure 6A. To elucidate whether miR‐612 can bind to 3′UTR of CCL28, we co‐transfected WT or Mut CCL28 3′UTR reporter systems and miR‐612 mimics into PC‐9 and 95D cells, and found that miR‐612 mimics markedly reduced the luciferase activities of WT CCL28 3′UTR, but displayed no effects on those of Mut CCL28 3′UTR (Figure 6B). According to the TCGA data, CCL28 was upregulated in pair‐matched and non‐paired NSCLC tissue samples (Figure S5A) and miR‐612 possessed a negative correlation with CCL28 expression in NSCLC (Figure S5B). In addition, increased expression of CCL28 was associated with pathological stage (*p* = 0.041) and lymph node infiltration (*p* = 0.003) in NSCLC (Table S4). Kaplan–Meier analysis revealed that the patients with CCL28‐high expression had a shorter survival as compared with those with CCL28‐low expression (Figure S5C,D). It was verified that miR‐612 inhibitors increased the mRNA and protein levels of CCL28 and abolished circSMOC1 knockdown‐caused CCL28 downregulation in PC‐9 and 95D cells (Figure 6C,D). Knockdown of CCL28 inhibited cell colony formation and invasion and counteracted miR‐612 inhibitor‐caused tumour‐promoting effects in PC‐9 and 95D cells (Figure 6E,F).

### 3.7 Knockdown of circSMOC1 Repressed In Vivo Tumorigenesis of NSCLC

**FIGURE 7:**
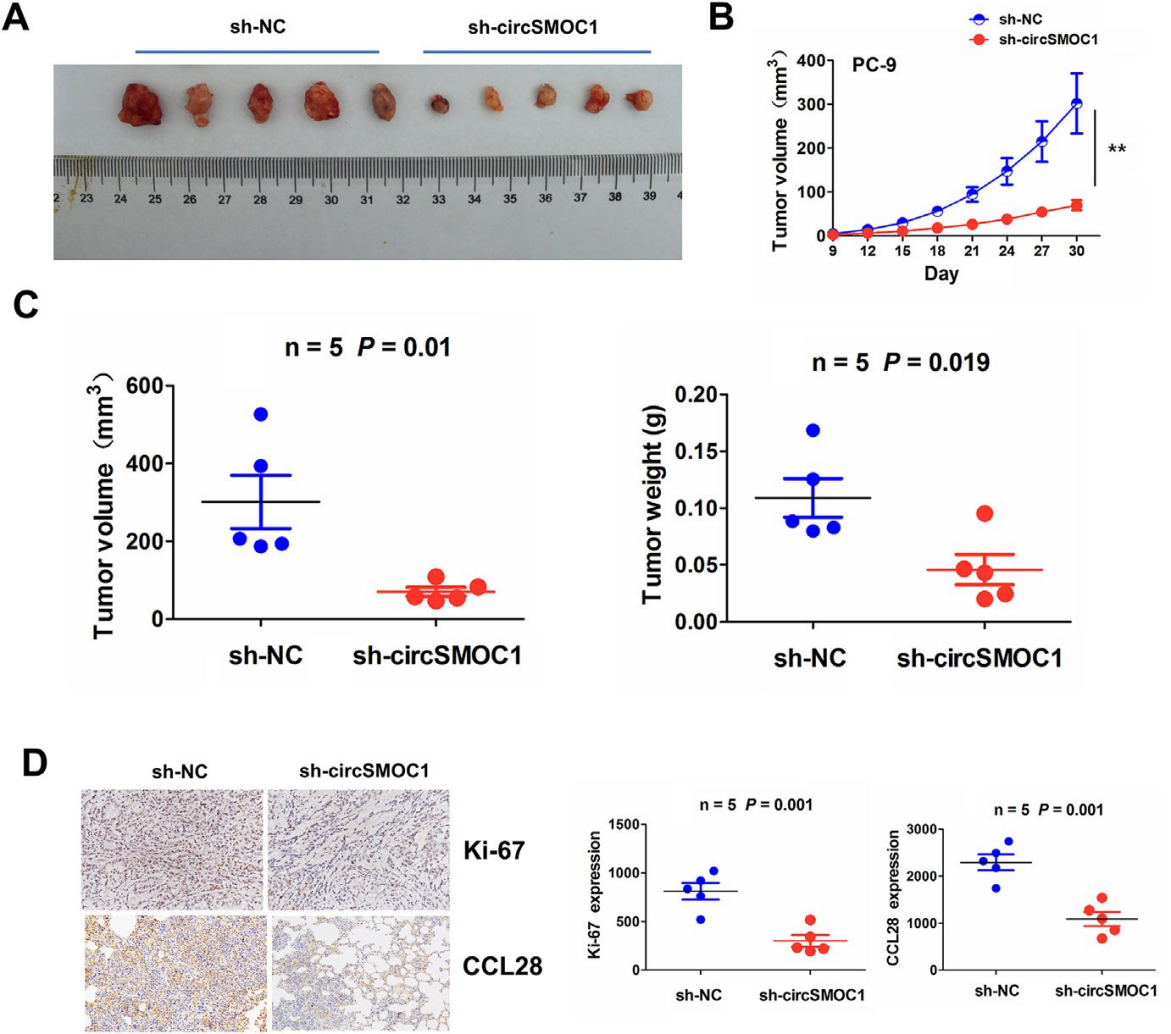
Knockdown of circSMOC1 suppressed in vivo NSCLC tumorigenesis. (A) Representative photographs of the subcutaneous xenograft tumours between sh‐circSMOC1 and sh‐NC groups. (B) Comparison of the tumour growth curve after treatment with sh‐circSMOC1 or sh‐NC transfected PC‐9 cells. (C) Comparison of the tumour volume and tumour weight between circSMOC1 and sh‐NC groups. (D) IHC analysis of the Ki‐67 levels and CCL28 protein expression between circSMOC1 and sh‐NC groups.

To rule out the effects of circSMOC1 on NSCLC tumorigenesis in vivo, PC‐9 cells stably transfected with sh‐NC or sh‐circSMOC1 were employed to subcutaneously inject into female nude mice (Figure 7A). The tumour growth curve demonstrated that the tumour grew slowly in the sh‐circSMOC1 group compared to the sh‐NC group (Figure 7B). The tumour volume was smaller and the tumour weight was lighter in the sh‐circSMOC1 group than the sh‐NC group (Figure 7C). IHC analysis showed that knockdown of circSMOC1 significantly lowered the Ki‐67 levels and CCL28 protein expression as compared with the sh‐NC group (Figure 7D).

## 4 Discussion

Accumulating studies have demonstrated that dysregulated circRNAs are associated with the prognosis and progression of NSCLC. Upregulation of circFGFR1 and hsa_circ_0003222 or downregulation of circNDUFB2 and circPTPRA is related to distant metastasis and poor prognosis in patients with NSCLC [8, 12, 13, 14]. Herein, we identified a novel hsa_circ_0032363 (circSMOC1) in NSCLC and found that elevated circSMOC1 expression was linked to TNM stage and poor survival in patients with NSCLC, suggesting that circSMOC1 may be a potential prognostic marker in NSCLC.

Previous findings indicated that circRNAs act as oncogenes or anti‐oncogenes in NSCLC. On the one hand, circFGFR1 and hsa_circ_0003222 facilitate cell proliferation, invasion and anti‐PD‐1 resistance in NSCLC [8, 12]. On the other hand, circNDUFB2, circPTPRA and hsa_circ_0008305 restrain epithelial‐mesenchymal transitioning and metastasis in NSCLC [13, 14, 15]. Individual study showed that circSMOC1 controls vascular calcification in vascular smooth muscle cells [30]. We herein found that knockdown of circSMOC1 suppressed cell proliferation, colony formation and invasion of NSCLC cells in vitro and in vivo, whereas ectopic circSMOC1 expression promoted these effects, indicating that circSMOC1 may be an oncogenic factor in NSCLC.

The m^6^A has been considered as the most prevalent modification in mRNAs and non‐coding RNAs [31] and participates in various malignancies, including NSLCL [22]. A series of studies unveil that m^6^A modification of circRNA influences cancer progression. For example, m^6^A modification of circMDK facilitates HCC tumorigenesis [32], m^6^A modification of circNSUN2 prompts colorectal liver metastasis [33] and m^6^A modification of circRNA‐SORE promotes sorafenib resistance in HCC [34]. Also, m^6^A‐modified circIGF2BP3 represses CD8^+^ T‐cell responses to promote immune evasion in NSCLC [35]. In the present study, we found that WTAP‐mediated m^6^A modification of circSMOC1 facilitated cell colony and invasion in NSCLC cells.

CircRNAs can act as miRNA sponges [8, 12, 14] and interact with IGF2BPs and TIF1γ [13, 15] implicated in NSCLC. CircSMOC1 has been reported to act as a miR‐874‐3p sponge, resulting in vascular calcification [30]. Herein, we discovered that circSMOC1 could be bound with the Ago2‐miR‐612 complex and decrease miR‐612 mRNA expression in NSCLC cells. CircETFA and hsa_circ_0001649 act as miR‐612 sponges to repress HCC tumorigenesis [20, 21]. These findings indicated that circSMOC1 may act as a sponge of miR‐612 to prompt NSCLC tumorigenesis.

MiR‐612 has been indicated as a tumour suppressor in multiple cancers [16, 17, 18, 19]. MiR‐612 is downregulated in LAC and suppresses cell proliferation by targeting FOXM1 [36]. In accordance, we found that miR‐612 expression was declined in NSCLC tissues and associated with a favourable prognosis in patients with NSCLC, exhibiting a negative correlation with circSMOC1 expression. MiR‐612 inhibitors enhanced cell proliferation and counteracted circSMOC1 knockdown‐caused anti‐proliferation effects. In addition, CCL28 is upregulated in multiple malignancies, including NSCLC [37] and pancreatic cancer [38] and blockade of CCL28 suppresses gastric cancer progression [39]. We herein identified that CCL28 was a direct target of miR‐612 and harboured poor survival in patients with NSCLC. miR‐612 inhibitors reversed circSMOC1 knockdown‐caused CCL28 downregulation. Knockdown of CCL28 inhibited cell colony formation and invasion and counteracted miR‐612 inhibitor‐caused tumour‐promoting effects in NSCLC cells. These findings indicated that WTAP could mediate m^6^A modification of circSMOC1, which acted as a sponge of miR‐612 to upregulate CCL28, contributing to NSCLC.

In short, our findings demonstrate that upregulation of circSMOC1 is associated with TNM stage and poor survival in patients with NSCLC. WTAP‐mediated m^6^A modification of circSMOC1 promotes the tumorigenesis and invasion of NSCLC cells by regulating the miR‐612/CCL28 axis, offering a potential therapeutic biomarker for NSCLC.

## Author Contributions

**Xun‐Xia Zhu:** data curation (equal). **Xiao‐Yu Chen:** data curation (equal). **Li‐Ting Zhao:** data curation (equal). **Xue‐Lin Zhang:** data curation (equal). **Yi‐Ou Li:** data curation (equal). **Xiao‐Yong Shen:** writing – review and editing (equal).

## Ethics Statement

The present study was approved by the Hospital's Protection of Human Subjects Committee.

## Consent

Consent for publication has been obtained from the patients.

## Conflicts of Interest

The authors declare no conflicts of interest.

## Supporting information


Appendix S1.

## Data Availability

All data generated or analysed during this study are included in this published article and its additional files.

## References

[1] R. L. Siegel , K. D. Miller , and A. Jemal , “Cancer Statistics, 2020,” CA: A Cancer Journal for Clinicians 70, no. 1 (2020): 7–30. 31912902 10.3322/caac.21590

[2] S. de Wit , E. Rossi , S. Weber , et al., “Single Tube Liquid Biopsy for Advanced Non‐Small Cell Lung Cancer,” International Journal of Cancer 144, no. 12 (2019): 3127–3137. 30536653 10.1002/ijc.32056

[3] C. Fang , L. Wang , C. Gong , et al., “Long Non‐Coding RNAs: How to Regulate the Metastasis of Non‐Small‐Cell Lung Cancer,” Journal of Cellular and Molecular Medicine 24, no. 6 (2020): 3282–3291. 32048814 10.1111/jcmm.15054 PMC7131947

[4] Y. Liu , X. Ao , W. Yu , Y. Zhang , and J. Wang , “Biogenesis, Functions, and Clinical Implications of Circular RNAs in Non‐Small Cell Lung Cancer,” Molecular Therapy–Nucleic Acids 27 (2021): 50–72. 34938606 10.1016/j.omtn.2021.11.013 PMC8645422

[5] L. S. Kristensen , M. S. Andersen , L. V. W. Stagsted , K. K. Ebbesen , T. B. Hansen , and J. Kjems , “The Biogenesis, Biology and Characterization of Circular RNAs,” Nature Reviews. Genetics 20, no. 11 (2019): 675–691. 10.1038/s41576-019-0158-7 31395983

[6] H. Liu , T. Lan , H. Li , et al., “Circular RNA CircDLC1 Inhibits MMP1‐Mediated Liver Cancer Progression via Interaction With HuR,” Theranostics 11, no. 3 (2021): 1396–1411. 33391541 10.7150/thno.53227 PMC7738888

[7] J. Zhang , H. Liu , L. Hou , et al., “Circular RNA_LARP4 Inhibits Cell Proliferation and Invasion of Gastric Cancer by Sponging miR‐424‐5p and Regulating LATS1 Expression,” Molecular Cancer 16, no. 1 (2017): 151. 28893265 10.1186/s12943-017-0719-3 PMC5594516

[8] P. F. Zhang , X. Pei , K. S. Li , et al., “Circular RNA circFGFR1 Promotes Progression and Anti‐PD‐1 Resistance by Sponging miR‐381‐3p in Non‐Small Cell Lung Cancer Cells,” Molecular Cancer 18, no. 1 (2019): 179. 31815619 10.1186/s12943-019-1111-2 PMC6900862

[9] L. He , C. Man , S. Xiang , L. Yao , X. Wang , and Y. Fan , “Circular RNAs' Cap‐Independent Translation Protein and Its Roles in Carcinomas,” Molecular Cancer 20, no. 1 (2021): 119. 34526007 10.1186/s12943-021-01417-4 PMC8442428

[10] Y. M. Sun , W. T. Wang , Z. C. Zeng , et al., “circMYBL2, a circRNA From MYBL2, Regulates FLT3 Translation by Recruiting PTBP1 to Promote FLT3‐ITD AML Progression,” Blood 134, no. 18 (2019): 1533–1546. 31387917 10.1182/blood.2019000802 PMC6839953

[11] N. Zhang , A. Nan , L. Chen , et al., “Circular RNA circSATB2 Promotes Progression of Non‐Small Cell Lung Cancer Cells,” Molecular Cancer 19, no. 1 (2020): 101. 32493389 10.1186/s12943-020-01221-6 PMC7268724

[12] C. Li , J. Zhang , X. Yang , et al., “hsa_circ_0003222 Accelerates Stemness and Progression of Non‐Small Cell Lung Cancer by Sponging miR‐527,” Cell Death & Disease 12, no. 9 (2021): 807. 34433810 10.1038/s41419-021-04095-8 PMC8387484

[13] B. Li , L. Zhu , C. Lu , et al., “circNDUFB2 Inhibits Non‐Small Cell Lung Cancer Progression via Destabilizing IGF2BPs and Activating Anti‐Tumor Immunity,” Nature Communications 12, no. 1 (2021): 295. 10.1038/s41467-020-20527-z PMC7804955 33436560

[14] S. Wei , Y. Zheng , Y. Jiang , et al., “The circRNA circPTPRA Suppresses Epithelial‐Mesenchymal Transitioning and Metastasis of NSCLC Cells by Sponging miR‐96‐5p,” eBioMedicine 44 (2019): 182–193. 31160270 10.1016/j.ebiom.2019.05.032 PMC6604667

[15] L. Wang , X. Tong , Z. Zhou , et al., “Circular RNA hsa_circ_0008305 (circPTK2) Inhibits TGF‐β‐Induced Epithelial‐Mesenchymal Transition and Metastasis by Controlling TIF1γ in Non‐Small Cell Lung Cancer,” Molecular Cancer 17, no. 1 (2018): 140. 30261900 10.1186/s12943-018-0889-7 PMC6161470

[16] Y. Liu , L. L. Lu , D. Wen , et al., “MiR‐612 Regulates Invadopodia of Hepatocellular Carcinoma by HADHA‐Mediated Lipid Reprogramming,” Journal of Hematology & Oncology 13, no. 1 (2020): 12. 32033570 10.1186/s13045-019-0841-3 PMC7006096

[17] Y. Liu , D. L. Liu , L. L. Dong , et al., “miR‐612 Suppresses Stem Cell‐Like Property of Hepatocellular Carcinoma Cells by Modulating Sp1/Nanog Signaling,” Cell Death & Disease 7, no. 9 (2016): e2377. 27685621 10.1038/cddis.2016.282 PMC5059880

[18] L. Sheng , P. He , X. Yang , M. Zhou , and Q. Feng , “miR‐612 Negatively Regulates Colorectal Cancer Growth and Metastasis by Targeting AKT2,” Cell Death & Disease 6, no. 7 (2015): e1808. 26158514 10.1038/cddis.2015.184 PMC4650731

[19] Y. Jin , X. Zhou , X. Yao , Z. Zhang , M. Cui , and Y. Lin , “MicroRNA‐612 Inhibits Cervical Cancer Progression by Targeting NOB1,” Journal of Cellular and Molecular Medicine 24, no. 5 (2020): 3149–3156. 31970934 10.1111/jcmm.14985 PMC7077537

[20] C. Lu , D. Rong , B. Hui , et al., “CircETFA Upregulates CCL5 by Sponging miR‐612 and Recruiting EIF4A3 to Promote Hepatocellular Carcinoma,” Cell Death Discovery 7, no. 1 (2021): 321. 34716323 10.1038/s41420-021-00710-x PMC8556257

[21] Y. Su , C. Xu , Y. Liu , Y. Hu , and H. Wu , “Circular RNA hsa_circ_0001649 Inhibits Hepatocellular Carcinoma Progression via Multiple miRNAs Sponge,” Aging 11, no. 10 (2019): 3362–3375. 31137016 10.18632/aging.101988 PMC6813922

[22] B. Zhou , F. Bie , R. Zang , et al., “RNA Modification Writer Expression Profiles Predict Clinical Outcomes and Guide Neoadjuvant Immunotherapy in Non‐Small Cell Lung Cancer,” eBioMedicine 84 (2022): 104268. 36116215 10.1016/j.ebiom.2022.104268 PMC9486036

[23] D. Jin , J. Guo , Y. Wu , et al., “m6A mRNA Methylation Initiated by METTL3 Directly Promotes YAP Translation and Increases YAP Activity by Regulating the MALAT1‐miR‐1914‐3p‐YAP Axis to Induce NSCLC Drug Resistance and Metastasis,” Journal of Hematology & Oncology 12, no. 1 (2019): 135. 31818312 10.1186/s13045-019-0830-6 PMC6902496

[24] D. Jin , J. Guo , Y. Wu , et al., “m6A Demethylase ALKBH5 Inhibits Tumor Growth and Metastasis by Reducing YTHDFs‐Mediated YAP Expression and Inhibiting miR‐107/LATS2‐Mediated YAP Activity in NSCLC,” Molecular Cancer 19, no. 1 (2020): 40. 32106857 10.1186/s12943-020-01161-1 PMC7045432

[25] Y. Shi , S. Fan , M. Wu , et al., “YTHDF1 Links Hypoxia Adaptation and Non‐Small Cell Lung Cancer Progression,” Nature Communications 10, no. 1 (2019): 4892. 10.1038/s41467-019-12801-6 PMC6814821 31653849

[26] K. Tsuchiya , K. Yoshimura , Y. Inoue , et al., “YTHDF1 and YTHDF2 Are Associated With Better Patient Survival and an Inflamed Tumor‐Immune Microenvironment in Non‐Small‐Cell Lung Cancer,” Oncoimmunology 10, no. 1 (2021): 1962656. 34408926 10.1080/2162402X.2021.1962656 PMC8366544

[27] Y. Chen , C. Peng , J. Chen , et al., “WTAP Facilitates Progression of Hepatocellular Carcinoma via m6A‐HuR‐Dependent Epigenetic Silencing of ETS1,” Molecular Cancer 18, no. 1 (2019): 127. 31438961 10.1186/s12943-019-1053-8 PMC6704583

[28] Z. X. Li , Z. Q. Zheng , P. Y. Yang , et al., “WTAP‐Mediated m6A Modification of lncRNA DIAPH1‐AS1 Enhances Its Stability to Facilitate Nasopharyngeal Carcinoma Growth and Metastasis,” Cell Death and Differentiation 29, no. 6 (2022): 1137–1151. 34999731 10.1038/s41418-021-00905-w PMC9177844

[29] W. Wei , J. Sun , H. Zhang , et al., “Circ0008399 Interaction With WTAP Promotes Assembly and Activity of the m6A Methyltransferase Complex and Promotes Cisplatin Resistance in Bladder Cancer,” Cancer Research 81, no. 24 (2021): 6142–6156. 34702726 10.1158/0008-5472.CAN-21-1518

[30] J. Ryu , N. Choe , D. H. Kwon , et al., “Circular RNA circSmoc1‐2 Regulates Vascular Calcification by Acting as a miR‐874‐3p Sponge in Vascular Smooth Muscle Cells,” Molecular Therapy–Nucleic Acids 27 (2021): 645–655. 35036071 10.1016/j.omtn.2021.12.031 PMC8752879

[31] Y. C. Yi , X. Y. Chen , J. Zhang , and J. S. Zhu , “Novel Insights Into the Interplay Between m6A Modification and Noncoding RNAs in Cancer,” Molecular Cancer 19, no. 1 (2020): 121. 32767982 10.1186/s12943-020-01233-2 PMC7412851

[32] A. Du , S. Li , Y. Zhou , et al., “M6A‐Mediated Upregulation of circMDK Promotes Tumorigenesis and Acts as a Nanotherapeutic Target in Hepatocellular Carcinoma,” Molecular Cancer 21, no. 1 (2022): 109. 35524319 10.1186/s12943-022-01575-z PMC9074191

[33] R. X. Chen , X. Chen , L. P. Xia , et al., “N6‐Methyladenosine Modification of circNSUN2 Facilitates Cytoplasmic Export and Stabilizes HMGA2 to Promote Colorectal Liver Metastasis,” Nature Communications 10, no. 1 (2019): 4695. 10.1038/s41467-019-12651-2 PMC6795808 31619685

[34] J. Xu , Z. Wan , M. Tang , et al., “N6‐Methyladenosine‐Modified CircRNA‐SORE Sustains Sorafenib Resistance in Hepatocellular Carcinoma by Regulating β‐Catenin Signaling,” Molecular Cancer 19, no. 1 (2020): 163. 33222692 10.1186/s12943-020-01281-8 PMC7681956

[35] Z. Liu , T. Wang , Y. She , et al., “N6‐Methyladenosine‐Modified circIGF2BP3 Inhibits CD8^+^ T‐Cell Responses to Facilitate Tumor Immune Evasion by Promoting the Deubiquitination of PD‐L1 in Non‐Small Cell Lung Cancer,” Molecular Cancer 20, no. 1 (2021): 105. 34416901 10.1186/s12943-021-01398-4 PMC8377850

[36] T. Shi , W. Sun , Y. L. Shi , Q. Wang , Z. X. Yan , and M. Zhang , “LncRNA OSER1‐AS1 Interacts With miR‐612/FOXM1 Axis to Modulate Gefitinib Resistance of Lung Adenocarcinoma,” American Journal of Translational Research 13, no. 3 (2021): 1365–1376. 33841662 PMC8014350

[37] S. Li , S. Liu , J. Xun , and F. Zhao , “Expression of CX3CL1 and CCL28 in Spinal Metastases of Lung Adenocarcinoma and Their Correlation With Clinical Features and Prognosis,” Journal of Healthcare Engineering 2022 (2022): 2580419. 35494513 10.1155/2022/2580419 PMC9050252

[38] J. Yan , P. Yuan , L. Gui , et al., “CCL28 Downregulation Attenuates Pancreatic Cancer Progression Through Tumor Cell‐Intrinsic and ‐Extrinsic Mechanisms,” Technology in Cancer Research & Treatment 20 (2021): 15330338211068958. 34939465 10.1177/15330338211068958 PMC8721394

[39] L. Ji , W. Qian , L. Gui , et al., “Blockade of β‐Catenin‐Induced CCL28 Suppresses Gastric Cancer Progression via Inhibition of Treg Cell Infiltration,” Cancer Research 80, no. 10 (2020): 2004–2016. 32156780 10.1158/0008-5472.CAN-19-3074

