# Supplementary material for: WTAP‐Mediated m6A Modification of circSMOC1 Accelerates the Tumorigenesis of Non‐Small Cell Lung Cancer by Regulating miR‐612/CCL28 Axis
Source: J Cell Mol Med. 2024 Dec 4;28(23):e70207. doi: 10.1111/jcmm.70207 (PMC11617116; doi:10.1111/jcmm.70207)
Supplement: Supplementary file 1 — Appendix S1. [file JCMM-28-e70207-s001.docx]

**Supplementary Figure legends**

**
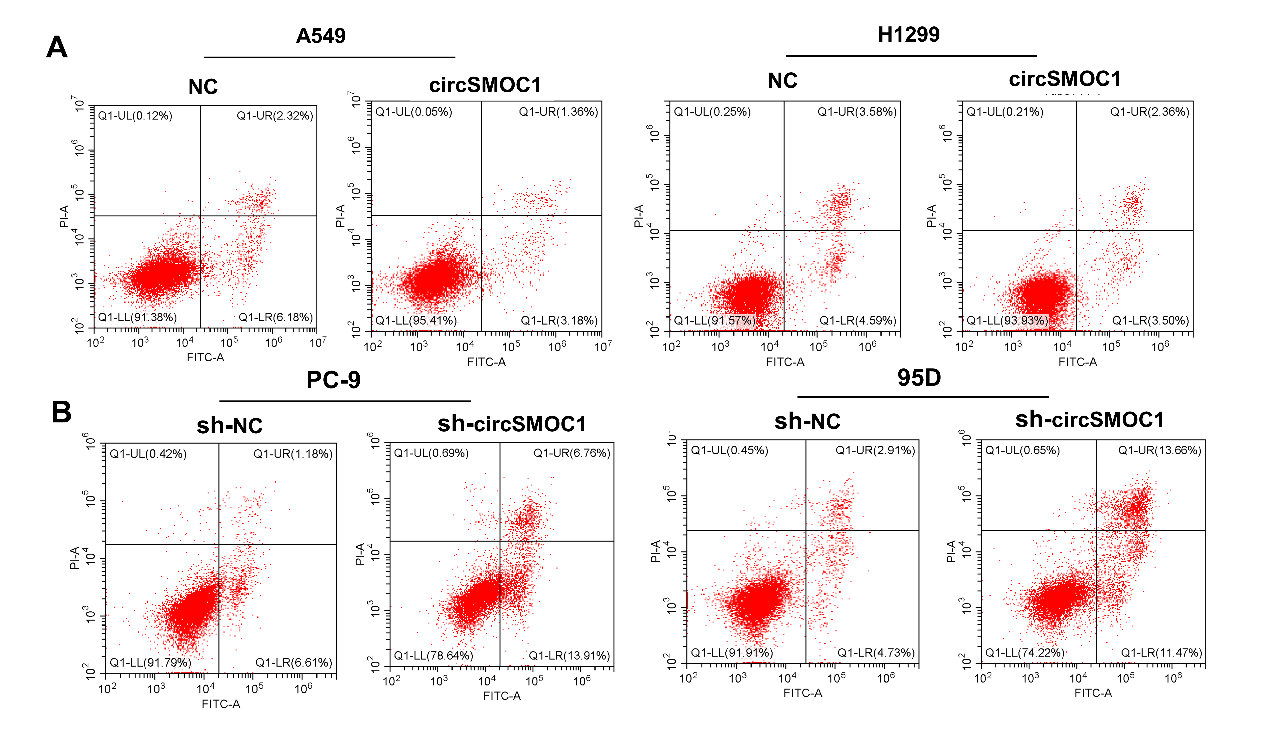
**

**Figure S1.** Flow cytometry analysis of the effects of circSMOC1 overexpression or knockdown on cell apoptosis in NSCLC cell lines.

**
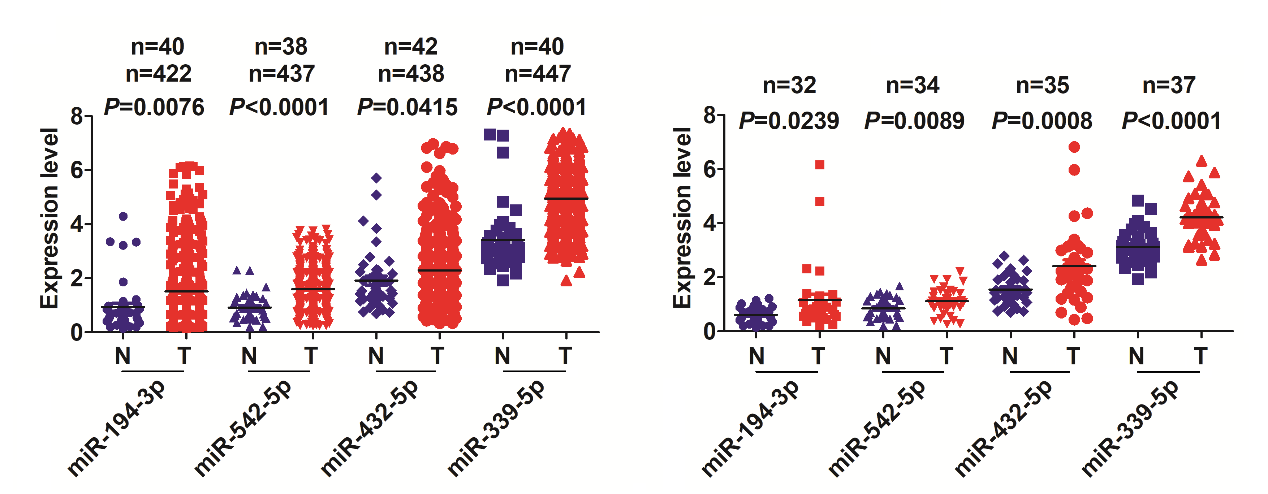
**

**Figure S2.** TCGA analysis of the expression levels of 4 miRNAs in pair-matched and non-matched NSCLC tissue samples.

**
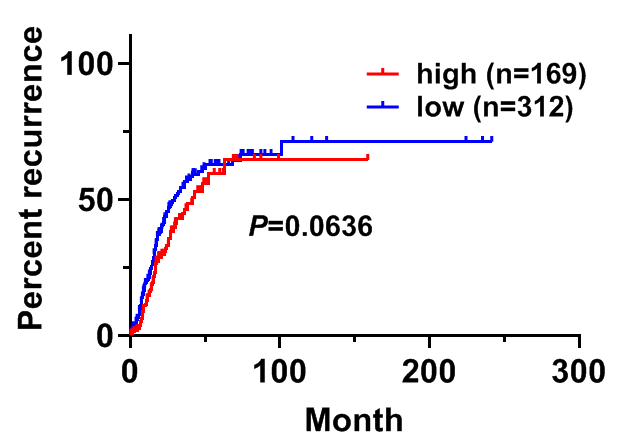
**

**Figure S3.** Kaplan-Meier analysis of the association of circSMOC1 expression with tumor recurrence in patients with NSCLC.

**
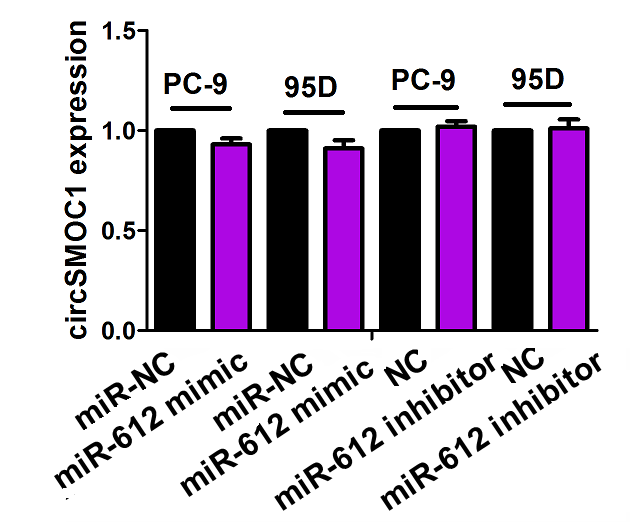
**

**Figure S4.** RT-qPCR analysis of the expression levels of circSMOC1 after transfection with miR-612 mimics or inhibitors in PC9 and 95D cells.

**
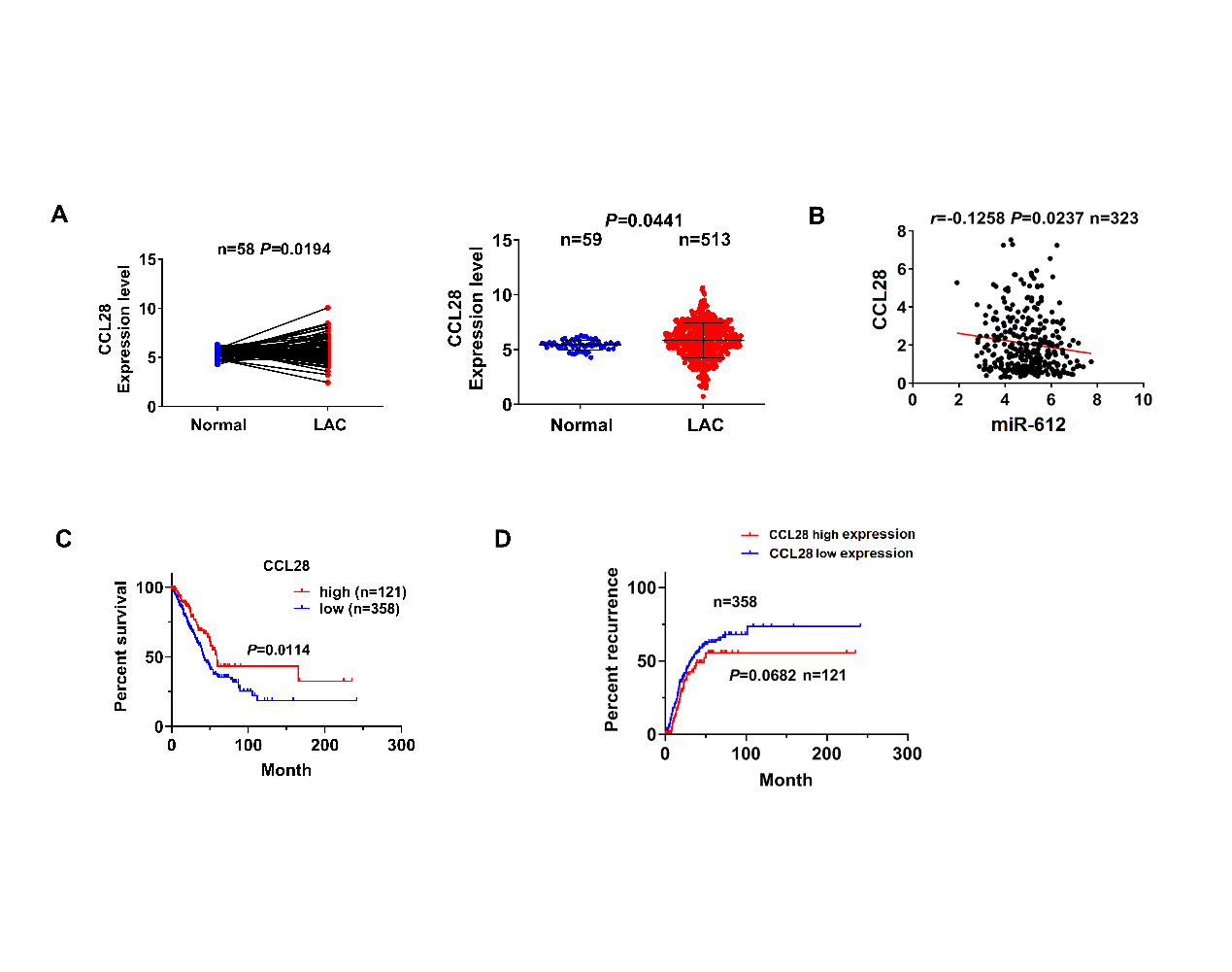
**

**Figure S5.** Kaplan-Meier analysis of the association of CCL28 expression with clinical prognosis in patients with NSCLC. (A) TCGA analysis of the expression levels of CCL28 in pair-matched and non-matched NSCLC tissues. (B) Pearson correlation analysis of the correlation of miR-612 with CCL28 expression in NSCLC tissue samples. (C) Kaplan-Meier analysis of the association of CCL28 with overall survival and tumor recurrence in patients with NSCLC.

**Supplementary Tables**

**Table S1.** The list of primer sequences

| Markers | Sense (5’-3’) | Antisense (5’-3’) |
| --- | --- | --- |
| CircSMOC1 | CTGGATGCTCTCACCACTGA | GTTGGGGCTGTGATTTCATC |
| SMOC1 | AGGTCCTACGAGTCCATGTGT | CACTGCACCTGGGTAAAGG |
| GAPDH | TCAAGAAGGTGGTGAAGCAGG | TCAAAGGTGGAGGAGTGGGT |
| miR-612 | GCAGGGCTTCTGAGCTCCTTAA | Universal reverse primer |
| U6 | CAGCACATATACTAAAATTGGAACG | ACGAATTTGCGTGTCATCC |
| WTAP | CAGCGATCAACTTGTTTTTCCTAAA | TCCTGGATAAGCATTCGACACTTC |
| CCL28 | TGCACGGAGGTTTCACATCAT | TTGGCAGCTTGCACTTTCATC |

**Table S2.** The association of circSMOC1 expression with clinicopathological

characteristics in patients with NSCLC

| Variables | Cases  (n) | circSMOC1 | | *P* value |
| --- | --- | --- | --- | --- |
|  |  | High | Low |  |
| Total | 80 | 46 | 34 |  |
| *Age (years)* |  |  |  |  |
| ≥60 | 40 | 24 | 16 |  |
| <60 | 40 | 22 | 18 | 0.653 |
| *Sex* |  |  |  |  |
| Male | 41 | 22 | 19 |  |
| Female | 39 | 24 | 15 | 0.479 |
| *Pathologic stage* |  |  |  |  |
| I-II | 41 | 24 | 17 |  |
| III-IV | 39 | 22 | 17 | 0.848 |
| *Tumor size (cm)* |  |  |  |  |
| ≥ 3 | 57 | 33 | 24 |  |
| < 3 | 23 | 13 | 10 | 0.911 |
| *TNM staging* |  |  |  |  |
| I-II | 38 | 16 | 22 |  |
| III-IV | 42 | 30 | 12 | 0.008 |
| *Lymph node metastasis* | l |  |  |  |
| Negative | 41 | 21 | 20 |  |
| Positive | 39 | 25 | 14 | 0.247 |

**Table S3** The correlation of miR-612 expression with clinicopathological

characteristics of LAC patients

| Variables | Cases  (n) | miR-612 | | *P* value |
| --- | --- | --- | --- | --- |
|  |  | High | Low |  |
| Total | 481 | 169 | 312 |  |
| *Age (years)* |  |  |  |  |
| ≥60 | 350 | 125 | 225 |  |
| <60 | 131 | 44 | 87 | 0.748 |
| *Gender* |  |  |  |  |
| Male | 220 | 67 | 153 |  |
| Female | 261 | 102 | 159 | 0.055 |
| *Pathological stage* |  |  |  |  |
| Ⅰ/Ⅱ | 378 | 146 | 232 |  |
| Ⅲ/Ⅳ | 103 | 23 | 80 | 0.002 |
| *T stage* |  |  |  |  |
| T1/T2 | 417 | 152 | 265 |  |
| T3/T4 | 64 | 17 | 47 | 0.159 |
| *N stage* |  |  |  |  |
| Negative | 312 | 124 | 188 |  |
| Positive | 169 | 45 | 124 | 0.005 |
| *M stage* |  |  |  |  |
| Negative | 318 | 104 | 214 |  |
| Positive | 163 | 65 | 98 | 0.131 |

**Table S4** The correlation of CCL28 expression with clinicopathological

characteristics of LAC patients

| Variables | Cases  (n) | CCL28 | | *P* value |
| --- | --- | --- | --- | --- |
|  |  | High | Low |  |
| Total | 481 | 287 | 194 |  |
| *Age (years)* |  |  |  |  |
| ≥60 | 350 | 213 | 137 |  |
| <60 | 131 | 74 | 57 | 0.405 |
| *Gender* |  |  |  |  |
| Male | 220 | 125 | 95 |  |
| Female | 261 | 162 | 99 | 0.263 |
| *Pathological stage* |  |  |  |  |
| Ⅰ/Ⅱ | 378 | 235 | 143 |  |
| Ⅲ/Ⅳ | 103 | 52 | 51 | 0.041 |
| *T stage* |  |  |  |  |
| T1/T2 | 417 | 255 | 162 |  |
| T3/T4 | 64 | 32 | 32 | 0.101 |
| *N stage* |  |  |  |  |
| Negative | 312 | 202 | 110 |  |
| Positive | 169 | 85 | 84 | 0.003 |
| *M stage* |  |  |  |  |
| Negative | 318 | 183 | 135 |  |
| Positive | 163 | 104 | 59 | 0.202 |
